# A multi-expert ensemble system for predicting Alzheimer transition using clinical features

**DOI:** 10.1186/s40708-022-00168-2

**Published:** 2022-09-03

**Authors:** Mario Merone, Sebastian Luca D’Addario, Pierandrea Mirino, Francesca Bertino, Cecilia Guariglia, Rossella Ventura, Adriano Capirchio, Gianluca Baldassarre, Massimo Silvetti, Daniele Caligiore

**Affiliations:** 1 Unit of Computer Systems and Bioinformatics, Department of Engineering, Università Campus Bio-Medico di Roma, Via Alvaro del Portillo 21, 00128 Rome, Italy; 2 Department of Psychology, Sapienza University, Piazzale Aldo Moro 5, 00185 Rome, Italy; 3 Computational and Translational Neuroscience Laboratory, Institute of Cognitive Sciences and Technologies, National Research Council (CTNLab-ISTC-CNR), Via San Martino della Battaglia 44, 00185 Rome, Italy; 4 IRCCS Fondazione Santa Lucia, Via Ardeatina, 306 and Via Del Fosso di Fiorano, 64, 00143 Rome, Italy; 5 AI2Life s.r.l., Innovative Start-Up, ISTC-CNR Spin-Off, Via Sebino 32, 00199 Rome, Italy; 6 Laboratory of Embodied Natural and Artificial Intelligence, Institute of Cognitive Sciences and Technologies, National Research Council (LENAI-ISTC-CNR), Via San Martino della Battaglia 44, 00185 Rome, Italy

**Keywords:** ADAS score, Cerebellar impairment, Clinical Dementia Rating Scale, Early diagnosis, Machine learning, Renal and genitourinary dysfunctions

## Abstract

Alzheimer’s disease (AD) diagnosis often requires invasive examinations (e.g., liquor analyses), expensive tools (e.g., brain imaging) and highly specialized personnel. The diagnosis commonly is established when the disorder has already caused severe brain damage, and the clinical signs begin to be apparent. Instead, accessible and low-cost approaches for early identification of subjects at high risk for developing AD years before they show overt symptoms are fundamental to provide a critical time window for more effective clinical management, treatment, and care planning. This article proposes an ensemble-based machine learning algorithm for predicting AD development within 9 years from first overt signs and using just five clinical features that are easily detectable with neuropsychological tests. The validation of the system involved both healthy individuals and mild cognitive impairment (MCI) patients drawn from the ADNI open dataset, at variance with previous studies that considered only MCI. The system shows higher levels of balanced accuracy, negative predictive value, and specificity than other similar solutions. These results represent a further important step to build a preventive fast-screening machine-learning-based tool to be used as a part of routine healthcare screenings.

## Introduction

Alzheimer’s disease (AD) is the most worldwide diffused neurodegenerative disorder affecting elders [1, 2]. It causes progressive impairments of memory, language, visuospatial skills, and executive functions together with progressive reduction of functional autonomy in daily life. Depression and apathy are also frequent in the early and middle stages of the disease, whereas neurological signs and motor impairments (e.g., dystonia, tremor) could emerge in later stages [3]. AD diagnosis is commonly based on the analysis of the patient’s medical history, clinical tests, clinical and neurological exams, and brain imaging data. Usually, the diagnostic evaluations are started when the first clinical symptoms begin to manifest. However, the progressive neurocognitive diseases underlying AD starts 10–15 years before deficits become clinically noticeable and disease is diagnosed [4]; therefore the diagnostic process takes usually place when severe damages of brain are already present [5–8].

The early, pre-clinical identification of individuals at high risk for developing AD is fundamental to provide a critical time window for early clinical management, treatment, and care planning, thus also reducing healthcare costs. Indeed, when supplied at the earlier pre-clinical disease phases, treatments could produce more important benefits [9, 10]. Moreover, during the pre-clinical stages lifestyle changes can be made that will slow or prevent AD development. For example, it could be possible to delay neurodegeneration by early modifying the exposure to certain risk factors such as hypertension, smoking, obesity, and diabetes [11, 12]. An early diagnosis and subsequent access to the proper services could help people live independently in their own homes for longer time and maintain a good quality of life for themselves, their families, and caregivers; also, it could allow people to plan and participate in their own legal, financial, and future support/care options and treatment when they still have the capacity to do so [13]. Early diagnosis gives patient’s relatives the time to adjust to the changes in function, mood, and personality that will occur when facing AD and their transition to a caregiver role, thus allowing them to feel more competent, acquire specific skills, reduce the stress and, as a consequence to suffer less from psychological problems such as anxiety and depression [14, 15].

Currently, MCI represents the earliest detectable stage of a potential ongoing progression toward AD. However, data indicate that only 20–40% of MCI individuals will convert to AD within 3 years from diagnosis [16, 17]. Researchers are investigating several promising biomarker candidates for AD onset anticipation, including brain imaging, proteins in cerebrospinal fluid (CSF), blood and urine tests, and genetic risk profiling [7, 8, 18]. Accuracy and timing are two critical aspects of these diagnostic approaches. While the literature shows that changes in biomarkers correlate with AD development, no single biomarker adequately predicts the conversion to AD of MCI patients and of healthy individuals, with an acceptable level of accuracy and well in advance with respect to the first manifestation of AD overt signs. Another critical aspect of current diagnostic approaches is that they require expensive tools (e.g., brain imaging), invasive clinical exams (amyloid-PET scan, CSF analysis), often also involving highly specialized personnel [13, 14].

Recent works support the use of Machine Learning (ML) tools into AD research and clinical practice to provide predictions with a certain degree of confidence, pivoting on information about the specific person (*personalized medicine*; [19–21]). These predictions support improved and more effective decision-making by researchers and clinicians [22, 23]. So far, many of these AI tools focus on predicting the AD conversion in MCI patients using different combinations of data from different sources, including genotyping, CSF biomarkers, brain imaging, demographic and clinical information, and cognitive performance ([18, 24–29]; see [30, 31], for recent reviews). Although some of these models could reach high levels of accuracy [32], consistency regarding what combination of features is more informative to predict AD as well as the translation into clinical practice are still lacking. One possible reason for this is that current AI algorithms still generally rely on expensive and invasive predictors, such as brain imaging or CSF biomarkers. As such, these studies only serve the purpose of a proof of concept, but do not represent a viable substitute of standard approaches with which they share application complexities and economic costs. To overcome these limitations, recent works proposed ML algorithms elaborating only *non-invasive* and *easy-to-collect* predictors (e.g., neuropsychological test scores, sociodemographic and clinical features, blood biomarkers) [20, 33].

In this paper, we developed, tested, and compared several ML algorithms and a weighted average rank ensemble ML system on the predictions provided by the various algorithms. The computer simulations show how the ensemble-based approach is a valuable AI tool for early detection of subjects at risk for developing AD. In particular, our system has four critical added values compared with similar approaches proposed in the literature. First, it extends the cohort of subjects by considering both healthy individuals and MCI patients drawn from the ADNI open dataset whereas previous studies mainly focused on MCI population; in this view, the system we proposed is aimed to provide a support for the early diagnosis in pre-clinical stages of AD in absence of MCI, that lacked in previous attempts. Second, it employs individuals whose diagnostic follow-up was available within 9 years after the baseline assessment. Most of the ML works proposed in literature focus on identifying biomarkers for early diagnosis starting from individuals whose diagnostic follow-up reached up to 3 years after the baseline assessment and mainly using a combination of neuroimaging, genetic and clinical data [34–36]. To the best of our knowledge, only few works investigated a greater time window to study the time point for conversion (from normal/MCI to AD) over 8 years using a combination of multi-scale genetic, neuroimaging and clinical data [37] or up to 5 years using MRI data [38]. The ML algorithm we proposed allows us to reach similar time windows (up to 9 years), but using only non-invasive and easily detectable clinical features. Third, it uses an optimized feature selection procedure to identify *only five* very easy-to-collect *predictors* based on neuropsychological test scores. This number of features is lower than that used by similar AI approaches [20, 33]. Finally, it shows higher balanced accuracy, negative predictive value, and specificity than previous similar approaches. Overall, these aspects make the AI system we propose here a clinically translatable early diagnostic tool to predict the conversion to AD within 9 years of healthy individuals and MCI patients, based on a low number of cost-effective, fast and easily collectable predictors.

## Materials and methods

### ADNI dataset

The data used in the preparation of this paper were obtained from the Alzheimer’s Disease Neuroimaging Initiative (*ADNI*) database (adni.loni.usc.edu). The ADNI was launched in 2003 as a public–private partnership, led by Principal Investigator Michael W. Weiner, MD. The primary goal of the ADNI project has been to test whether serial magnetic resonance imaging (MRI), positron emission tomography (PET), other biological markers, and clinical and neuropsychological assessment can be combined to measure the progression of MCI and early AD. For the selection and extraction of the dataset, the data were imported into a *MySql* database. In order to obtain the best possible dataset, the imported data were checked, cleaned from errors and missing data (such as checking for null values), and organized for the next stage of processing to eliminate redundant or incomplete data and select high-quality data. The database consisted of several tables, one table for each file downloaded from ADNI. The cleaned and selected data were collapsed into a single table through *SQL* and exported to a single *CSV* file for subsequent processing.

### Cohort chosen for the study

For this study, we employed data from *n* = 525 participants, using identification numbers (RID; each uniquely assigned to a subject). The data were downloaded on Jan 30, 2021. We first manually select 69 features (i.e., test scores) from the ADNI database based on their availability and facility administration in the clinical context (most are already routinely assessed in clinical practice, see below). We used data chosen from ADNI 1 first exam date, then we extracted the data on the same patients (based on RID) in ADNI 2 collected at last 5 years apart. We indicate each feature with the same name used in ADNI. In particular, for each recording related to each patient, we combined demographic measures (sex, age, marital status, handedness, education) (Table 1), data from different neuropsychological tests such as:

- American National Adult Reading Test (ANARTERR) which is used to estimate premorbid verbal levels of intelligence in dementing individuals [39].
- Boston Naming Test (BNTTOTAL) which is used to assess naming ability [40].
- Category Fluency Test (CATANIMSC,CATANINTR,CATANPERS) which is a test used to measure ability to spontaneously generate a set of semantically related words in 1 min [39].
- Clinical Dementia Rating (CDR) which is a five-point semi-structured interview between the patient and a reliable informant (e.g., caregivers) designed to stage the severity of dementia considering different aspects (memory (CDMEMORY), orientation (CDORIENT), judgment and problem solving (CDJUDGE), community affairs (CDCOMMUN), home and hobbies (CDHOME), personal care (CDCARE), global summary (CDGLOBAL)) [41, 42].
- Clock Drawing Test (CLOCKSCOR, COPYSCOR) in which subjects draw a clock and set the hands to 10 after 11 [43]
- Cognitive Subscale Alzheimer’s Disease Assessment Scale (ADAS14) (85 points including Q4 (Delayed Word Recall) and Q14 (Number Cancellation)) which is composed of two parts, the noncognitive subscale and the cognitive subscale, and returns a measure index of global cognition [44, 45].
- Geriatric Depression Scale (GDTOTAL) which is a self-report assessment used to identify mood changes in elderly patients [46].
- Neuropsychiatric Inventory Questionnaires, a short version of the Neuropsychiatric Inventory (NPISCORE), which is a brief self-administered questionnaire [47].
- Mini Mental State (MMSCORE) which is a brief questionnaire measuring the global cognitive impairment [48].
- Rey Auditory Verbal Learning Test (RAVLT_forgetting_bl, RAVLT_immediate_bl, RAVLT_learning_bl, RAVLT_perc_forgetting_bl) that is a cognitive test used to evaluate verbal learning and memory [49].
- Trail Making Test (TRAASCOR,TRABSCOR), a test with two parts, the first is relative to psychomotor process, the second is relative to cognitive flexibility [50].

And other data such as:

- Family history (FHQMOM = mother, FHQDAD = father, FHQSIB = siblings) relative to dementia.
- Comorbidity with Parkinson’s disease (DXPARK).
- Medical history diseases (psychiatric (MHPSYCH), neurological (MH2NEURL), head problem (MH3HEAD), cardiovascular (MH4CARD), respiratory (MH5RESP), hepatic (MH6HEPAT), dermatological (MH7DERM), musculoskeletal (MH8MUSCL), endocrine-metabolic (MH9ENDO), gastrointestinal (MH10GAST), hematopoietic-lymphatic (MH11HEMA), renal (MH12RENA), allergies (MH13ALLE), alcohol abuse (MH14ALCH), smoking (MH16SMOK), malignancy (MH17MALI), other kind of problems (MH19OTHR)).
- Physical and neurological exams (general appearance (PXGENAPP), head general aspect (PXHEADEY), neck (PXNECK), chest (PXCHEST), heart (PXHEART), abdomen (PXABDOM), peripheral vascular (PXPERIPH), musculoskeletal (PXMUSCUL), visual (NXVISUAL) and auditory (NXAUDITO) impairment, tremor (NXTREMOR), cranial nerves (NXNERVE), motor strength (NXMOTOR), Cerebellar—Finger to Nose (NXFINGER), Cerebellar—Heel to Shin (NXHELL), sensory (NXSENSOR), deep tendon reflexes (NXTENDON), plantar reflexes (NXPLANTA), gait (NXGAIT)).

**Table 1:** Subjects composition

Variable	
Male	292
Female	233
Age (years)	[55 to 90]; mean 73
Married	397
Divorced	43
MCI	241 (EMCI = 55; LMCI = 186); conv. to AD 104
NT	284 (SMC = 59); conv. to AD 20
Right hand	474
Left hand	51
Education (years)	[6 to 20]; mean 16
Evaluation time lap (years)	[5 to 13]; mean 9

EMCI: early MCI; LMCI: late MCI; SMC: subjective memory complaints; NT: normotypical

### Data pre-processing

A logistic lasso regression method was applied as supervised feature selection method with *L*1 regularization [51, 52]. We used this regularization method because it has the effect of keeping in the final model only the most significant features, in particular the method forces the coefficients of less discriminating features toward zero. Furthermore, to face the dataset unbalance we applied the class weights technique modifying the training algorithm to take into account the different numerosity of the classes, giving different weights to the majority and minority classes [53]. Before applying the method all data were subject to standardization (null mean and standard deviation equal to one) in order to homogenize the feature scale. The classification used two classes, ‘convert to Alzheimer’ and ‘non convert to Alzheimer’, as indicated by the last test of each participant of her/his dataset after the evaluation time lapse. As shown in Eq. 1, the logistic regression estimates a binary decision function where the logit can be modeled as a linear function of features:

1 $$\begin{aligned} \log\Big (\frac{p_\beta (x_i)}{1-p_{\beta }({\textbf {x}}_i)}\Big )=\beta _0+\sum {\textbf {x}}_{i,j}^\text{T}\beta _j, \end{aligned}$$

where “*i*” is the index of sample, “*q*” the index of feature, and $\beta _0$ is the intercept and $\beta _j$ is coefficient of *j*th feature and $p_{\beta }(x_i)=P(Y=1\vert {\textbf {x}}_i)$ with *Y*$\in \{0,1\}$. The L1 penalty parameter is introduced into the model to reduce the estimates of the regression coefficients towards zero and to set some of them against the maximum likelihood estimates:

2 $$\begin{aligned} {\hat{\beta }}= -L(\beta _0,\beta _j)+\uplambda \Vert \beta \Vert _1, \end{aligned}$$

where *L* is the log-likelihood function and $\uplambda$ is the regularization parameter. We also perform standard statistical data analysis (Tables 2 and 3).

**Table 2:** Descriptive statistics for the ordinal data of all subjects (525)

Feature	Min	Max	Mean	Std. dev.
**ADAS14**	1	40	15.79	8.64
AGE	55	90	73.27	6.54
ANARTERR	0	47	10.78	8.39
BNTTOTAL	11	30	27.61	2.83
CATANIMSC	6	38	19.39	5.35
CATANINTR	0	6	0.06	0.46
CATANPERS	0	13	0.74	1.25
CDCARE	0	1	0.01	0.12
CDCOMMUN	0	1	0.07	0.18
CDGLOBAL	0	0.5	0.23	0.25
CDHOME	0	1	0.07	0.18
CDJUDGE	0	1	0.16	0.25
**CDMEMORY**	0	2	0.25	0.29
CDORIENT	0	2	0.10	0.22
CLOCKSCOR	1	5	4.59	0.71
COPYSCOR	0	5	4.81	0.50
GDTOTAL	0	6	1.13	1.29
MMSCORE	24	30	28.44	1.58
NPISCORE	0	17	1.25	2.23
PTEDUCAT	6	20	16.28	2.74
RAVLT_forgetting_bl	− 3	15	4.31	2.51
RAVLT_immediate_bl	13	70	40.72	11.24
RAVLT_learning_bl	0	11	5.15	2.58
RAVLT_perc_forgetting_bl	− 37.5	100	47.18	31.46
TRAASCOR	13	150	36.01	15.35
TRABSCOR	32	300	90.92	47.92

In bold the feature selected by the optimized procedure used for the features selection

We selected the best parameter $C=\frac{1}{\uplambda }$ weighting the effect of the regularization of the feature selection algorithm through a tenfold cross-validation grid search on a range of the parameter described by the Python function logspace (0.1, 4, 20) that generates a row vector of 20 logarithmically spaced points between decades $10^{0.1}$ and $10^{4}$. Small values of *C* imply a strong regularization which leads to find simple models underfitting the data. Large values of *C* imply a low regularization which allows a higher complexity of the model overfitting the data.

**Table 3:** Descriptive statistics for the nominal data of all subjects (525)

Feature	Mode	Min	Max
DXPARK	0	0	1
FHQDAD	0	0	2
FHQMOM	0	0	2
FHQSIB	1	0	1
MHPSYCH	0	0	1
MH2NEURL	0	0	1
**MH3HEAD**	1	0	1
MH4CARD	1	0	1
MH5RESP	0	0	1
MH6HEPAT	0	0	1
MH7DERM	0	0	1
MH8MUSCL	1	0	1
MH9ENDO	0	0	1
MH10GAST	0	0	1
MH11HEMA	0	0	1
**MH12RENA**	0	0	1
MH13ALLE	0	0	1
MH14ALCH	0	0	1
MH16SMOK	0	0	1
MH17MALI	0	0	1
MH19OTHR	0	0	1
NXVISUAL	1	1	2
NXAUDITO	1	1	2
NXTREMOR	1	1	2
NXNERVE	1	1	2
NXMOTOR	1	1	2
NXFINGER	1	1	2
**NXHEEL**	1	1	2
NXSENSOR	1	1	2
NXTENDON	1	1	2
NXPLANTA	1	1	2
NXGAIT	1	1	2
PTGENDER	1	1	2
PTHAND	1	1	2
PTMARRY	1	1	5
PXGENAPP	1	1	2
PXHEADEY	1	1	2
PXNECK	1	1	2
PXCHEST	1	1	2
PXHEART	1	1	2
PXABDOM	1	1	2
PXPERIPH	1	1	2
PXMUSCUL	1	1	2

In bold the feature selected by the optimized procedure used for the features selection

The features selection process used a tenfold cross-validation method. To this purpose, we divided the data into tenfolds (sets). Out of the tenfolds, nine sets were used for training while the remaining set was used for testing; this process was then repeated 10 times using a different fold for each test. The score used in the test directed to isolate the best *C* was based on the average recall of the two classes. This process led to find $C=2.019$ as the regularization value leading to the maximum scores. To select only the most relevant features and implement a tighter dimensionality reduction on the method with the best parameter *C*, we selected only features with a coefficient greater than 0.5. In this way, we apply a stricter feature selection by selecting only those features that have an odds ratio greater than $e^{0.5}=0.64$ and so a odds to have a discriminating impact greater of $\%60$, in fact $(1.64-1)=1.64$.

**Fig. 1:**
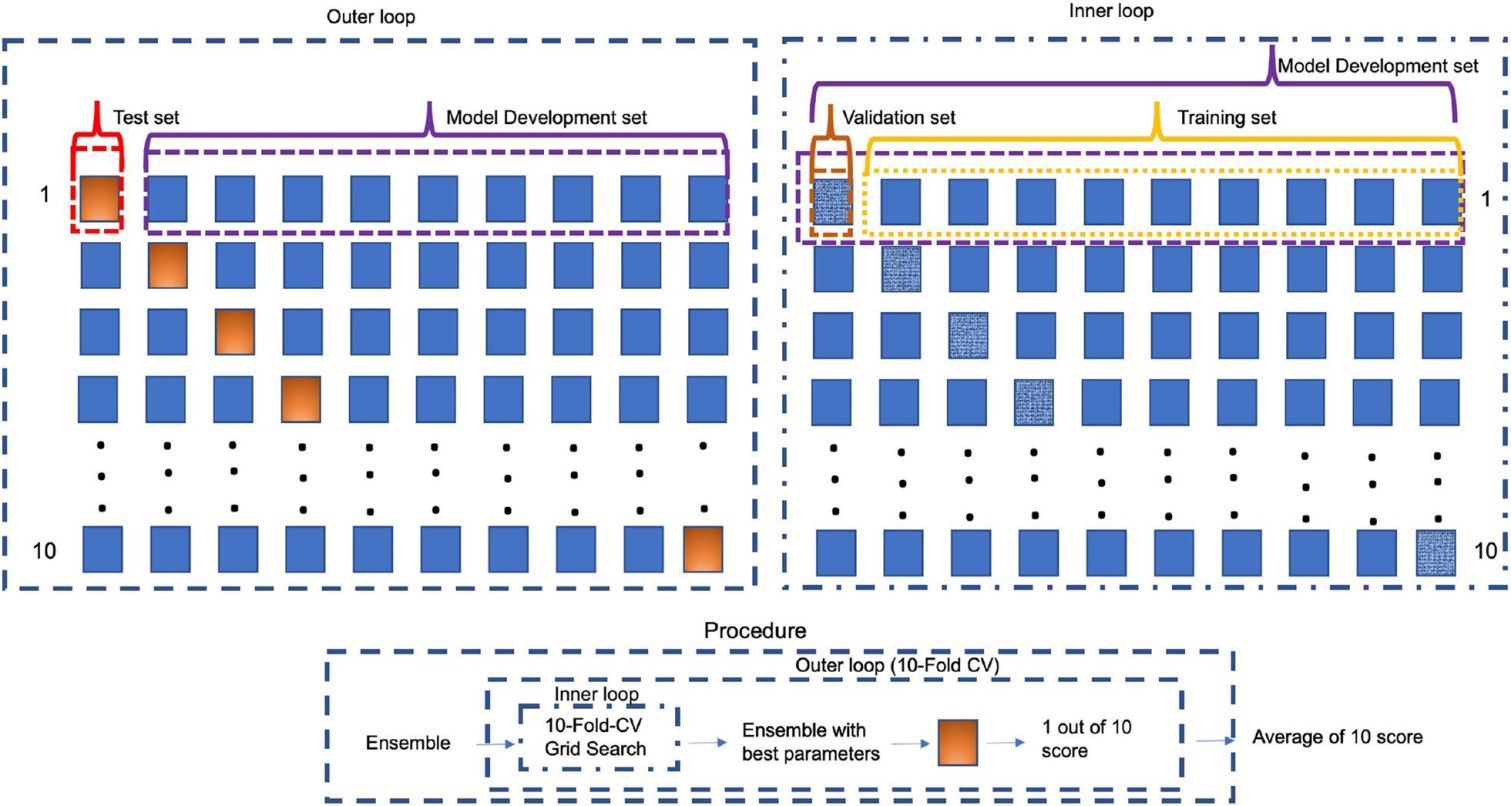
Nested tenfold cross-validation (CV) procedure for model development and evaluation. In the outer CV loop (on top left), the dataset was partitioned into the ‘Model Development Set’ and ‘Test Set’. In the inner CV loop (on top right), the ‘Model Development Set’ was further partitioned into the ‘Training Set’ and ‘Validation Set’. The inner loop was composed of tenfold cross-validation Grid Search with the aim of obtaining the best parameters for each of the three classifiers assembled. On the bottom of figure, the procedure for one single iteration of the outer CV loop is graphed in diagram form

### Classification model

To face the binary classification problem we used an Multi-Experts Ensemble model (MEE) composed of a random forest [54], a Neural Net [55], and a Support Vector Machine [56]. Ensemble methods usually produce more accurate solutions than single models do. This approach obtains the final prediction in the test phases by averaging the predictions of three classifiers with the hard majority voting rule. In developing the assembled classifier in addition to preliminary results, we chose a combination of classifiers that would allow us to analyze three different feature representation spaces based on the main learning paradigms Decision Tree (RF), Kernel Method (SVM) and Deep Learning (NN). To train the system and evaluate its performance we used the 10-Repeated-Nested-10-Fold-Cross-Validation procedure. In particular, we used this method to select the hyperparameters of each model of the ensemble classifier, and to achieve the average performance of ensemble method [57, 58]. In this way, we avoid model overfitting and optimistically biased estimates of model performance.

This procedure was composed of two cross-validation (CV) loops, each implementing a tenfold stratified CV:

- In the outer CV loop designed to obtain an unbiased estimate of model performance, the dataset was partitioned into the ‘Model Development Set’ and the ‘Test Set’. This is schematized in the upper left part of Fig. 1;
- For each iteration of outer CV loop, an entire inner CV loop was performed. The inner CV loop was designed to select the optimal hyperparameters for the final model through a Grid Search technique with the accuracy on validation set as selection score [59]. The ‘Model Development Set’ was further partitioned into the ‘Training Set’ and ’Validation Set’. This is schematized in the upper right part of Fig. 1.

The above reported whole procedure was repeated 10 times to verify the robustness of the method and the low influence of the initial random choice of the samples in the tenfolds. The completed procedure is outlined in the lower part of Fig. 1. Table 4 shows details of the three models forming the ensemble as well as the ranges of the hyperparameters used for the grid search. The neural network we used was composed of one hidden layer with rectified linear units, and one output layer with 2 logistic units. The network size was set small due to the small size of the input patterns and to avoid overfitting.

**Table 4:** Hyperparameters of the three models forming the MEE, and their range used by the grid search method

	Models	Hyperparameters	Range
Ensemble proposed	Neural Net	Optimizer	{‘SGD’, ‘Adagrad’, ‘Adadelta’, ‘Adam’, ‘Adamax’, ‘Nadam’}
		Batch size	{10, 20, 40, 60, 80, 100}
		Epochs	{10, 50, 100}
		Number of hidden units	{2:2:50}
	Random Forest	Max depth	{5, 20, 50, 80, 110}
		Min samples for leaf	{3, 4, 5, 10]}
		Min samples for split	{8, 10, 12, 24, 32}
		Number of estimators	{30, 200, 300, 1000}
	Support Vector Machine	C parameter	{0.1,1, 10, 100}
		$\gamma$ parameter	{1, 0.1, 0.01, 0.001}
		Kernel	{‘linear’, ‘poly’, ‘rbf’, ‘sigmoid’}

## Results and discussion

All tests were developed in Python and used Scikit-learn and Keras as main libraries [60]. The first key result of our study comes from the optimized procedure used for the features selection. This isolated only five critical features (on 69 initially considered, see Sect. 2.2) for very early prediction of AD development: one from the CDR, one from the ADAS14, two from the medical history questionnaires (MH3HEAD for Head, Eyes, Ears, Nose and Throat problems, and MH12RENA for Renal-Genitourinary problems), and one from neurological exams (NXHELL Cerebellar Heel to Shin, for cerebellar dysfunction). CDR and ADAS14 are two of the most common tests used in clinical practice for AD diagnosis and evaluation. CDR is a global clinical scale to evaluate different cognitive performances through six specific subscales with established diagnostic and severity-ranking utility and used for research in epidemiological studies and clinical trials as well as for patient evaluation in clinical practice [41, 61]. In particular, the optimized feature selection procedure described in Sect. 2 identified the CDR memory subscale (CDMEMORY) as one of the most relevant features to predict AD development. This result agrees with data suggesting that early episodic memory impairments related to pathologic changes in the hippocampus and entorhinal cortex are common AD initial symptoms. Several data show that memory impairment could be a good predictor for the conversion of MCI in AD [62], and memory dysfunctions could appear up to 7 years before AD diagnosis [63]. Aside from CDMEMORY, the features selection procedure underlined how the ADAS14 score is another critical feature to predict AD development. This result is in line with the crucial role that ADAS14 plays as a gold standard for assessing the efficacy of antidementia treatments [44, 45].

The optimized feature selection procedure also evidenced how some impairments (apparently) far from traditional AD neurodegenerative processes, like head injury and renal and cerebellar dysfunctions, could be critical features to predict AD development. Several studies support this result. Head injuries could lead to long-term problems with cognitive functioning and increase the risk of cognitive decline, which progresses faster in older individuals who suffer from head injuries than in those who did not [64, 65]. In addition, traumatic brain injuries could contribute to AD development, and if present in early or middle life, could increase the risk of late-life AD occurring [66–69].

There is complex pathophysiology of cognitive decline in chronic kidney disease (see [70] for a review). Kidney dysfunctions could contribute to impairments in semantic, episodic, and working memory. Furthermore, a lower estimated glomerular filtration rate at baseline was associated with a more rapid rate of cognitive decline [71].

Genetic mutations in Presenilin-1 protein have been described both in patients with cerebellar ataxia and in early AD onset [7, 72, 73]. In addition, MCI patients show lower cerebellar grey matter volumes compared with age-matched individuals, and total cerebellar grey matter volume decreases as the disease evolves. Furthermore, the decrease of cerebellar grey matter volume appears to be a predictable pattern to cerebellar grey matter atrophy in AD. This cerebellar impairment first affects the vermis and the posterior lobe and then the anterior lobe (for a review see [74]). Overall, these results suggest framing AD according to a *system-level perspective*, where the interactions between brain–body dysfunctions could be critical for early diagnosis [19, 75].

The second interesting result of the present study comes from the analysis of the predictive power of the ML algorithms. The first row of Table 5 reports the performance achieved by the proposed system in terms of sensitivity, specificity, accuracy, negative predictive value, balanced accuracy, and *F*1-score. To develop a complete comparison, we tested and optimized other classifiers belonging to different learning paradigms, including a Multi-Layer Perceptron (MLP) as a neural network, a k-Nearest Neighbor (kNN) as an instance-based classifier, a Support Vector Machine (SVM) as a kernel machine, a Naive Bayes (NB) as a Bayesian classifier, a Decision Tree (DT) as a non-parametric classifier model, a Logistic Regression (LR) as a probabilistic regression model for classification, and finally a Random Forest (RF) and a Adaptive Boosting (AdaBoost) as a classification ensemble. All these systems were tested and trained with the same technique described in Sect. 2.4.

**Table 5:** Performance of the ML algorithms

Classifier models	Sensitivity (%)	Specificity (%)	Precision (%)	NPV (%)	BA (%)	*F*1-score
MEE proposed	73.5	88.3	68.3	91.5	80.9	70.8
AdaBoost	54.2	93.3	73.8	87.0	73.7	62.5
MLP	76.3	78.4	54.4	91.6	77.4	63.5
NB	48.5	90.0	61.1	85.4	69.4	54.1
DT	59.1	87.0	66.0	87.5	73.1	62.4
KNN	52.8	93.5	73.4	87.4	74.6	61.4
LR	75.0	79.5	55.2	91.3	77.3	63.6
RF	68.6	86.0	63.1	89.9	77.3	65.7
SVM	65.6	89.0	66.9	89.4	77.3	66.2

Sensitivity: ratio between the AD converter subjects correctly labeled by the algorithm and all subjects that actually converted; Specificity: ratio between the non-AD converter subjects correctly labeled by the algorithm and all subjects that have not actually converted; Precision: ratio between the correctly AD converter subjects labeled by the algorithm and the AD converters; Negative predictive value (NPV): the proportion of predicted negatives which are real negatives. It reflects the probability that a predicted negative is a true negative; Balanced accuracy (BA): the average between sensitivity and specificity; *F*1-score: the harmonic average of the sensitivity and precision

For all systems, the values of the hyperparameters that were most frequently found to be optimal during the optimization procedure and the average score obtained with the grid search are reported in Table 6, whereas their performance is reported in the remaining rows of Table 5.

**Table 6:** Reports for each method the average score obtained during Grid Search and values of hyperparameters most frequently selected during *k*-fold nested-cross-validation

	Classifier models	Average score Accuracy on validation set (%)	Best hyperparameters
Ensemble proposed	NN	78.5	Optimizer: Adam
			Batch size: 60
			Epochs: 100
			Number of hidden units: 32
	RF	81.7	Max depth: 80
			Min samples for leaf: 3
			Min samples for split: 12
			Number of estimators: 100
	SVM	83.5	*C*: 0.1;
			$\gamma$: 1;
			Kernel: radial basic function
	AdaBoost	80.6	Algorithm: SAMME
			Learning rate: 0.1
			Number of estimators: 250
	MLP	72.3	Activation: identity
			Batch size: 20
			Epochs: 80
			Optimizer: Adam
			Number of hidden units: 16
	NB	–	–
	DT	78.3	Criterion of split: Gini
			Max depth: 2
			Min samples for leaf: 5
			Split method: best
	KNN	54.2	Distance metric: manhattan
			Number of neighbors: 19
	LR	78.6	*C*: 0.0885
			Penalty: L1
			Solver: Newton-cg

Table 5 shows that the ensemble solution produces, on average, better predictive performances than the other algorithms we tested. In addition, compared with similar works that used only non-invasive and easily detectable clinical features [20], our system has a better negative predictive power. In particular, it can predict if a subject *will not develop* AD with higher performances in terms of specificity, negative predictive value, and balanced accuracy. This result could be critical for developing fast-screening protocols. The other metrics (sensitivity, precision, and *F*1-score) are similar to those obtained with similar approaches proposed in literature. These works, however, used a substantially larger number of features, prediction windows up to 3 years, and focused only on MCI patients. The ensemble-based ML algorithm proposed here can predict AD development within 9 years from first overt signs not only in MCI patients, but also in healthy individuals.

Despite these encouraging results, future improvements of our approach, for example in terms of generalization, could be obtained by enhancing the heterogeneity of the training set, and including data from different countries (e.g., Asia and Europe). In this way, it would be possible to detect different lifestyle and epigenetic elements that could act as risk or protective factors in AD development.

## Conclusion

The current approaches for AD diagnosis often require invasive and expensive tools (e.g., brain imaging) and highly specialized personnel, and start at a time-point where the disorder has already caused severe brain damages, the underlying neuropathology may be less sensitive to treatment and the clinical signs are apparent [5, 6, 8]. A critical challenge of our years is to develop an artificial tool able to detect AD onset with many years of advances in order to limit or stop symptoms altogether ([36] for a review). Several works try to answer this question by integrating different aspects of AD pathophysiology, such as neuroimaging, plasma biomarkers, and genetic data [76–79]. The proposed approaches could be very accurate, but also expensive. This aspect could limit their use since another challenge is to make early diagnosis accessible to all [80]. Moreover, most of the ML works proposed in literature focus on identifying biomarkers for early diagnosis starting from individuals whose diagnostic follow-up reached up to 3 years [34, 35].

This article proposes an ensemble-based ML algorithm for predicting AD development within 9 years from first overt signs and using only five non-invasive and easily detectable clinical tests. The results we obtained represent a first important step towards building a preventive fast-screening machine-learning tool usable as part of a routine healthcare visit. In this way, it could help to identify individuals that might develop AD at an early pre-clinical stage and in cost-effective ways without raising undue anxiety associated with attending a specialized clinic [13].

## Data Availability

Data used in preparation of this article were obtained from the Alzheimer’s Disease Neuroimaging Initiative (ADNI) database (adni.loni.usc.edu), which is easily available for download from the Laboratory of Neuroimaging (LONI) website to the research public. As such, the investigators within the ADNI contributed to the design and implementation of ADNI and/or provided data, but did not participate in analysis or writing of this report. A complete listing of ADNI investigators can be found at http://adni.loni.usc.edu/wp-content/uploads/how_to_apply/ADNI_Acknowledgement_List.pdf.
